# Delay discounting without decision-making: medial prefrontal cortex and amygdala activations reflect immediacy processing and correlate with impulsivity and anxious-depressive traits

**DOI:** 10.3389/fnbeh.2015.00280

**Published:** 2015-10-29

**Authors:** Vera U. Ludwig, Corinna Nüsser, Thomas Goschke, Dina Wittfoth-Schardt, Corinde E. Wiers, Susanne Erk, Björn H. Schott, Henrik Walter

**Affiliations:** ^1^Division of Mind and Brain Research, Department of Psychiatry and Psychotherapy, Charité Universitätsmedizin BerlinBerlin, Germany; ^2^Berlin School of Mind and Brain, Humboldt Universität zu BerlinBerlin, Germany; ^3^Division of Medical Psychology, University of BonnBonn, Germany; ^4^Department of Psychology, Technische Universität DresdenDresden, Germany; ^5^Institute of Neuroradiology, Hannover Medical SchoolHannover, Germany; ^6^NICA—NeuroImaging and Clinical ApplicationsHannover, Germany; ^7^Leibniz-Institute for NeurobiologyMagdeburg, Germany

**Keywords:** reward, delay discounting, impulsivity, individual differences, fMRI, amygdala, prefrontal cortex

## Abstract

Humans value rewards less when these are delivered in the future as opposed to immediately, a phenomenon referred to as delay discounting. While delay discounting has been studied during the anticipation of rewards and in the context of intertemporal decision-making, little is known about its neural correlates in the outcome phase (during reward delivery) and their relation to personality. Personality traits that have been associated with increased delay discounting include impulsivity and, potentially, anxious-depressive traits. Here we performed functional magnetic resonance imaging (fMRI) in 72 healthy participants while they carried out a monetary incentive delay (MID) task with a delay manipulation. In sixty percent of the experimental trials, participants won rewards that differed in magnitude (0.05€, 0.50€ or 1€) and delay until delivery (immediately, 10 days, or 100 days). A factor analysis on questionnaires yielded two factors reflecting Impulsivity and Anxiety/Depression, which we used to examine potential relationships between personality and delay discounting. When winning a reward, medial prefrontal cortex (mPFC) activation was higher for immediate compared to delayed rewards. Moreover, amygdala activation correlated with reward magnitude for immediate but not for delayed rewards. Amygdala activation to winning immediate rewards was higher in more impulsive participants, while mPFC activation to winning immediate rewards was higher in more anxious-depressed participants. Our results uncover neural correlates of delay discounting during reward delivery, and suggest that impulsivity and subclinical anxious-depressive traits are related to stronger neural responses for winning immediate relative to delayed rewards.

## Introduction

Animals and humans typically prefer immediate over delayed rewards, even if the latter are of larger absolute value (Rachlin and Green, 1972; Mischel and Underwood, 1974; Ainslie, 1975; Mazur, 1988; Dshemuchadse et al., 2013). The phenomenon of *delay discounting* is evident in daily decisions like food choices or smoking behavior and poses a challenge to health education. To elucidate neural mechanisms of delay discounting, several studies have adapted delay discounting paradigms to functional magnetic resonance imaging (fMRI; for recent reviews, see Peters and Büchel, 2011; van den Bos and McClure, 2013). For this purpose, most previous studies worked with experimental designs that involved decisions between two rewards that differed in magnitude and delay until delivery (McClure et al., 2004, 2007; Kable and Glimcher, 2007, 2010). While this approach has yielded important insights into the neural basis of delay discounting, it has two limitations. First, decision-making is likely to engage several different cognitive processes, most prominently the valuation of rewards and action selection processes (see Rangel et al., 2008; Liu et al., 2012), and the commonly employed choice paradigms do not allow one to distinguish between these subprocesses. Second, framing effects, as proposed by cognitive delay discounting theories (Trope and Liberman, 2003; Zauberman and Lynch, 2005), may occur because valuation of one option is always affected by an available alternative (Marjorie, 1993).

Because the decision component of delay discounting may cognitively resemble other decision-making processes, the unique properties of delay discounting might actually lie in the valuation component. Valuation automatically occurs whenever humans encounter stimuli in their environment (Lebreton et al., 2009). Few studies so far have been specifically directed at the dissociation of valuation and decision-making components within delay discounting. In a two-phase paradigm employed by Liu et al. (2012), participants first evaluated two options (one immediate, one delayed; i.e., valuation phase), and made their decisions only in the second phase. In the valuation phase only, activation in ventromedial prefrontal cortex (vmPFC), ventral striatum (VS), and posterior cingulate cortex correlated with value, while the decision-making phase engaged lateral prefrontal cortices. However, given the study design participants may already have engaged in decision-making during the first phase of a trial. The safest way to exclude decision-making all together is to only present one option at a time, for example by using a monetary incentive delay (MID) task (Knutson et al., 2001a). Luo et al. (2009) adapted the MID task to study delay discounting (see also Luo et al., 2011). At the beginning of each trial, a cue predicting either an immediate reward or a delayed reward (e.g., $28 in 4 months) was presented, and participants could win the reward by responding to a target. The authors found that brain regions implicated in value processing (e.g., putamen, anterior insula) responded more strongly to immediate vs. delayed rewards, even though the immediate and the delayed rewards were preference-matched. Since only one reward was anticipated at a time, activation differences could only reflect valuation or motivational processes, but not decision-making.

Luo et al. (2009) focused on the effects of delay discounting during reward anticipation. One may argue, however, that valuation does not only occur during anticipation, but also in the outcome phase when participants have overcome the uncertainty inherent in the anticipation phase. Knutson et al. (2001b) showed that, in healthy young adults, anticipation and delivery of rewards engage largely distinct neural processes, with the VS/nucleus accumbens (NAcc) responding to cues signaling an upcoming reward, while the delivery of a previously anticipated reward is primarily associated with activation of regions in the medial prefrontal cortex (mPFC; see also Knutson et al., 2003; Schott et al., 2007). Given the role of the mPFC in coding stimulus value and personal preferences (Knutson et al., 2005; Ludwig et al., 2014; Lin et al., 2015), it seems important to consider the outcome phase when investigating the valuation component of delay discounting. In the present study, we used a variant of the MID task, but, unlike Luo et al. (2009), who only reported the neural correlates of delay discounting during reward anticipation, we focused our analyses on the outcome phase.

While high degrees of delay discounting can be observed in a number of neuropsychiatric disorders like drug addiction (Kirby and Petry, 2004; Mitchell et al., 2005), pathological gambling (Petry, 2001; Alessi and Petry, 2003), ADHD (Scheres et al., 2006, 2008), or Cluster B personality disorders (e.g., Petry, 2002), delay discounting is also subject to considerable interindividual variability within the healthy population (Odum, 2011). Despite the heterogeneity of models of personality (e.g., Costa and McCrae, 1992a; Cloninger et al., 1993), a few personality traits are widely accepted, and their corresponding constructs can be found in most established models. Among those traits, impulsivity in particular has repeatedly been associated with behavioral and neural measures of delay discounting. Impulsivity can be broadly defined as the tendency to act on arising impulses without much thinking or planning, and it is likely to be a complex, multifaceted construct (Patton et al., 1995; Evenden, 1999). A particularly strong preference for immediate compared to delayed rewards (i.e., high delay discounting) is thought to be a key feature of impulsivity. Indeed, tasks of intertemporal decision-making have consistently demonstrated a positive relationship between impulsivity and delay discounting (but see Reynolds et al., 2006; de Wit et al., 2007; Mobini et al., 2007; Koff and Lucas, 2011), in line with the increased delay discounting rates in psychiatric disorders associated with high impulsivity (e.g., Petry, 2002). At the neural level, Sripada et al. (2011) showed that more impulsive participants exhibited reduced anterior mPFC activation during decisions that involved one immediate option as compared to decisions involving only delayed options (see also Hariri et al., 2006; Luhmann et al., 2008; Jimura et al., 2013).

Another important construct widely accepted as a personality trait is the (subclinical) presence of anxious and depressive symptoms, both of which contribute to concepts like neuroticism (NEO-Five Factor Inventory [NEO-FFI]; Costa and McCrae, 1992a) or harm avoidance (Temperament and Character Inventory [TCI]; Cloninger, 1994). While impulsivity has rather consistently been linked to high delay discounting rates, a potential relationship between anxious-depressive traits and delay discounting has thus far received little attention. One study has found that individuals high on social anxiety demonstrate higher delay discounting rates than those low on social anxiety (Rounds et al., 2007). More generally, psychiatric disorders involving anxiety or depression have been associated with altered reward processing (Elman et al., 2005; Tremblay et al., 2005; Hopper et al., 2008; Sailer et al., 2008; Aupperle and Paulus, 2010). It is therefore conceivable that subclinical anxious-depressive traits may affect neural or behavioral manifestations of delay discounting, although it is not straightforward to predict the direction of the correlation: While models of approach vs. avoidance might predict, if at all, lower delay discounting in anxious or depressed individuals, the study by Rounds et al. (2007) suggests stronger delay discounting effects in more anxious-depressed individuals.

In the present study we investigate the neural underpinnings of delay discounting without decision-making in the outcome phase and further assess how individual differences in impulsivity and anxious-depressive traits relate to those neural processes. In our MID-paradigm participants could win rewards that differed in both magnitude and delay until delivery. The paradigm also included a behavioral measure: on each trial participants had to carry out a simple classification task in order to have the chance to gain a reward. Reaction times (RTs) during this task served as an indicator of participants’ incentive motivation to obtain each specific reward.

Neuroanatomically, we focused on the VS, mPFC, and amygdala because these regions have been commonly associated with reward and emotional processing (Knutson et al., 2001a,b, 2003; Hommer et al., 2003; Heekeren et al., 2007; Plichta et al., 2009; Schardt et al., 2010); and activation in mPFC (Sripada et al., 2011) and in the VS (McClure et al., 2004) have specifically been linked to the processing of the immediacy of rewards (Table 1). We hypothesized: (i) a main effect of reward magnitude on the VS and mPFC (in line with previous findings) and (ii) a main effect of delay on the VS, the mPFC, and the amygdala in that these regions would show increased activation during winning immediate (compared to delayed) rewards (“immediacy effect”). We further hypothesized (iii) an interaction of delay and reward magnitude that was thought to reflect a stronger magnitude effect for immediate (compared to delayed) rewards.

**Table 1:** **Regions of interest (ROIs)**.

ROI	Type and origin of the ROI	Motivation for selecting the ROI
mPFC	ROI from the Stanford atlas of functional ROIs (Shirer et al., 2012)	- implicated in the outcome phase of MID tasks (Knutson et al., 2001b; Knutson et al., 2003)
		- associated with impulsivity (e.g., Sripada et al., 2011)
VS	Combined anatomical and literature-based ROI (Zweynert et al., 2012)	- a key region for reward processing (Heekeren et al., 2007; Staudinger et al., 2011)
		- associated with impulsivity (e.g., Jimura et al., 2013)
Amygdala	Anatomical ROI from the AAL atlas as implemented in the WFU Pickatlas (Maldjian et al., 2003)	- a key region for reward processing and emotional processing (e.g., Hommer et al., 2003; Plichta et al., 2009; Schardt et al., 2010; Patin and Hurlemann, 2011),
		- associated with impulsivity (e.g., Shao et al., 2013).

*An illustration of the location and extent of the ROIs can be found in Supplementary Table S1*.

We further aimed to assess to what extent individual differences in impulsivity and anxious-depressive traits might correlate with the differences of neural responses to immediate vs. delayed rewards. With respect to impulsivity, we hypothesized that (iv) more impulsive participants would show stronger activation in the VS or amygdala—and possibly reduced activation in the mPFC—when winning immediate (compared to delayed) rewards, as compared to less impulsive participants (e.g., Mobini et al., 2007). Regarding anxious-depressive traits, we also expected to find (v) a correlation between these traits and neural effects of immediacy during outcome although we had no specific hypotheses about the direction.

Behaviorally, we expected participants to show shorter RTs to both larger and immediate rewards compared to smaller and delayed rewards, respectively (i.e., main effects of magnitude and delay, as well as potentially an interaction of magnitude × delay). Moreover, we expected that the effect of delay would be associated with longer RTs in more impulsive individuals, while we had no directional hypothesis with respect to RTs in participants with anxious-depressive traits.

## Materials and Methods

### Participants

Our study cohort consisted of seventy-two young (mean age = 23.10, range 20–29, SD = 2.23) healthy, right-handed, volunteers (35 female) recruited from the campus community of the University of Bonn, Germany. Eight additional participants were excluded from data analysis due to poor quality of the fMRI data and/or excessive movement during MRI acquisition (*n* = 4), incidental pathological findings in T1-weighted MR images (*n* = 3), and a defective anatomical image (*n* = 1). In all analyses involving questionnaires, we included only the 62 participants with complete data sets. In all other analyses, we included all 72 participants (62 with complete datasets + 10 with partly missing questionnaire data). All participants had normal or corrected-to-normal vision and were native speakers of German. None of the participants reported any current or past neurological, psychiatric, or medical illness (including alcohol or illegal drug abuse), or use of medication affecting cerebral blood flow or brain metabolism. Thirty-three of the participants were smokers. The study was approved by the University of Bonn Ethics Committee, and written informed consent was obtained from each participant in accordance with the Declaration of Helsinki. Participants received financial compensation for their participation.

### Behavioral Paradigm and Experimental Design

**Figure 1:**
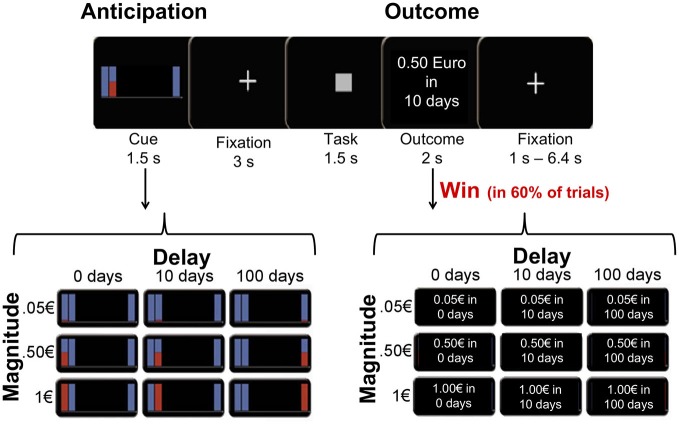
**Task and design.** Participants were presented with a cue signaling the magnitude of an upcoming reward (0.05€, 0.50€ or 1€) and the delay to delivery (0, 10, or 100 days). After correctly responding to a simple task (classifying squares and triangles), participants were rewarded with the anticipated amount in 60% of trials, and they were informed about the outcome at the end of each trial.

Participants carried out a variant of the MID task, allowing the assessment of blood-oxygen-level dependent (BOLD) responses to reward anticipation and outcome (Figure 1). Each participant was first provided with both written and verbal task instructions and performed a short training version of the experiment outside the scanner to minimize learning effects during the experiment and to ensure compliance with the procedure.

At the beginning of each trial, one of nine possible rewards (3 amounts [1€, 0.50€, 0.05€] × 3 payoff times [immediately, in 10 days, in 100 days]) was presented as an abstract image cue for 1.5 s (anticipation; Figure 1). The reward cue was followed by a delay period during which a fixation cross was presented for 3 s. After the delay, a symbol was presented on the screen, and participants were instructed to classify it as a square or a triangle by pressing a button with their left or right index finger (counterbalanced across participants). Squares and triangles were presented in randomized order. RTs were recorded as a behavioral measure of incentive motivation. If the button press was carried out correctly and within 1.5 s after target onset, participants had a 60% chance of winning the anticipated reward. Participants were informed that their RTs had no influence on their chance of winning a reward, as long as the response occurred within 1.5 s. Reward delivery (win trial) or omission (omission trial) was indicated by a feedback, which was presented for another 2 s (outcome period). In win trials, the outcome screen confirmed the anticipated reward and delay in written words, while in omission trials the outcome screen stated “no win”. Omission trials were included in the experimental design to prevent habituation and decreasing attention, and to minimize the correlation between the anticipation phase and the win-outcome phase. Each trial lasted 8 s. Trials were presented in a randomized order and separated by a variable inter-trial interval (range 1–6.4 s). The experiment consisted of two runs, each lasting about 23 min with a total number of 234 trials (26 of each condition).

All rewards gained in the “today” win trials were paid to the participants in cash immediately after the experiment. The gains of the “in 10 days” or “in 100 days” win trials were transferred to their bank accounts either 10 or 100 days after the experiment. The *Presentation* software package (Neurobehavioral Systems, CA, USA) was used for stimulus presentation, synchronization of the stimulus display to fMRI data acquisition, and recording of participants’ behavioral responses. Stimuli were presented using video goggles (Nordic-Neuro-Lab, Norway). Behavioral responses were recorded via two fiber-optics response pads.

### Questionnaire Measures

We used data from several well-established questionnaires testing anxious-depressive and/or impulsive personality traits. Specifically, we included scores from Spielberger’s State-Trait Anxiety Inventory (trait score only; STAI-T; German version by Spielberger et al., 1970; Laux et al., 1981), from Beck’s Depression Inventory (BDI; Beck et al., 1996; German version by Hautzinger et al., 2000), and from the NEO-FFI (Costa and McCrae, 1992b) in the German translation by Borkenau and Ostendorf (1993), which includes 60 items. The NEO-FFI contains the subscales neuroticism, extraversion, openness to experience, agreeableness, and conscientiousness. Furthermore, we included data from the TCI by Cloninger (1994; German version by Richter et al., 1999). This scale includes 240 items, but here we were only interested in the three subscales novelty seeking, harm avoidance, and reward dependence, corresponding to 99 items only. Finally, we used the total score of the Barratt Impulsiveness Scale (BIS-11), which consists of 30 items and assesses general impulsivity (BIS-11; Patton et al., 1995).

### Factor Analysis of Questionnaire Data

The considerable overlap of the constructs assessed with currently established personality questionnaires like the NEO-FFI or the TCI has been noted by several authors (De Fruyt et al., 2000; Aluja and Blanch, 2011). Because we had no *a priori* hypothesis with respect to which questionnaire would best reflect the intermediate phenotypes of interest and in order to avoid a large number of multiple comparisons, we carried out a factor analysis on the questionnaire data to determine factors that reflected impulsive and axious-depressive traits in our variables (for similar approaches, see Whiteside and Lynam, 2003; Aluja and Blanch, 2011). We initially entered the variables BIS-11 total score, BDI, the 5 NEO-factors, STAI-T, and the 3 TCI-variables. Kaiser-Meyer-Olkin (KMO) measures of sampling adequacy (see Kaiser, 1970) for NEO openness, NEO agreeableness, and TCI reward dependence were lower than 0.50. Therefore, these variables were excluded from the factor analysis. With the remaining 8 variables included, KMO-value for the set of variables was 0.73, indicating that our data were well-suited for factor analysis. Factors were orthogonalized using direct oblimin rotation (delta = 0). Factors with eigenvalues greater than 1 were retained (Kaiser, 1960). Factor scores were calculated for each participant using the regression method. All behavioral and questionnaire data were analyzed using SPSS 19.0 (Chicago, IL, USA) software.

### Analysis of the Behavior on the Visual Discrimination Task

First, participants’ response accuracy was calculated to ascertain that participants were attentive and motivated during the experiment. Median instead of mean accuracy was calculated across participants because the accuracy data were not normally distributed.

Second, we calculated median RTs for all correct button presses in the visual discrimination task per condition and participant. Median instead of mean RTs were used on the single-subject level because medians are robust to outliers and non-normal distribution. At the group level, RTs did not violate assumptions of a normal distribution (Kolmogorov-Smirnov tests: all *p* > 0.10, Bonferroni-corrected). We therefore analyzed these RTs in all participants (*n* = 72) using a mixed ANOVA with the within-subject factors delay (0 days, 10 days, 100 days) and magnitude (0.05€, 0.50€, 1€). Because the within-subject factors had more than two levels, Greenhouse-Geisser correction for non-sphericity was applied to the degrees of freedom. In order to determine if the factor scores Anxiety-Depression and Impulsivity were systematically related to RTs on the task, in a second step we included these factor scores as covariates (for the 62 participants with complete datasets).

### fMRI Data Acquisition and Preprocessing

Functional MRI was acquired using a 1.5T Avanto MRI system (Siemens, Erlangen, Germany). T2*-weighted MR images were acquired with a gradient-echo echoplanar imaging (EPI) sequence (TR = 2000 ms, TE = 50 ms, flip angle = 90°, ascending order). Twenty-three axial slices were collected (thickness 3.3 mm; gap 1.1 mm; field of view 210 mm) using a 4-channel head coil. Slices were oriented parallel to the anterior commissure—posterior commissure line so that they covered the mesolimbic and prefrontal regions of interest (ROI). We also collected a high-resolution T1-weighted magnetization-prepared rapid gradient echo (MP-RAGE) anatomical image (160 sagittal slices covering the whole head; thickness 1 mm; gap 0.5 mm; field of view 256 mm).

Functional MRI data were analyzed using Statistical Parametric Mapping (SPM8, Wellcome Trust Center for Neuroimaging, University College London, London, UK^1^) running on Matlab 7.11.0 (MathWorks, Natick, MA, USA). T2*-weighted EPIs were corrected for acquisition delay and spatially registered to the first acquired image (without reslicing). Each participant’s high-resolution anatomical image was co-registered to the mean EPI obtained from realignment and was then segmented into gray matter, white matter, and CSF, using the segmentation algorithm provided by SPM. (*Note*: For three participants, the anatomical image was not obtained in the course of this study. In these cases, we used an anatomical image from the respective participant obtained in the course of a different study, but within 3 months of study time). The transformation parameters obtained from segmentation were used as normalization parameters for transformation of the EPIs into a standard stereotactic reference frame (Montreal Neurological Institute, MNI; voxel size = 3 × 3 × 3 mm). Normalized images were smoothed using an isotropic Gaussian kernel of 8 mm full-width half-maximum. Low frequency drifts were removed using a high pass filter with a cut-off of 128 s during the analysis. Intrinsic autocorrelations were corrected using a restricted maximum likelihood (ReML) algorithm using an autoregressive model of 1st order (AR1).

### fMRI Data Analysis

Statistical analysis was performed using a two-stage mixed effects model. At the first stage, general linear model (GLM)-based analyses of brain activity patterns were performed for each participant. For each of the two runs, the GLM included nine regressors for the experimental conditions in the anticipation period (duration: 4.5 s), one regressor for the visual discrimination task (duration: 1.5 s), nine regressors for the “win” outcome periods (duration: 2 s), nine regressors for the “omission” period (duration: 2 s), and one regressor for outcomes of missed or incorrect responses (duration: 2 s). For all regressors, stick functions at stimulus onset were convolved with the canonical hemodynamic response function (HRF) and down-sampled for each scan. Additionally, the six rigid-body transformation parameters obtained from realignment were included in the model for each session, plus a single constant representing the mean over scans. Model estimation was carried out with a ReML fit, and contrasts of interest were computed over the resulting parameter estimates.

At the second level, contrasts of parameter estimates were submitted to flexible-factorial random effects models. Specifically, we computed two separate second level ANOVA models: one for the anticipation phases, and one for the win-outcome phases. Omission-outcomes were not analyzed further. Nine first-level contrasts for anticipation (all nine conditions against baseline) or nine first-level contrasts for win-outcomes (all nine conditions against baseline) were entered as regressors into these two separate analyses. The reason for contrasting gain outcomes with baseline rather than with omission outcomes was that omissions of expected outcomes would elicit a negative prediction error (Abler et al., 2005) that would depend on the expected value (EV; the product of gain magnitude and probability; see Knutson et al., 2005) and thus on individual task performance. Because we did not include a jitter between cues and feedback stimuli, our design could not account completely for a certain degree of correlation between the anticipation phase and the win outcome phase, but we aimed to minimize this problem by keeping the average gain probability at approximately 60%, and by modeling first degree serial autocorrelations during data analysis (see below).

In these flexible factorial analyses, we specified the factors *delay* (3 levels, independence not assumed, equal variances assumed), *magnitude* (3 levels, independence not assumed, equal variances assumed), and *subject* (72 levels, accounts for task-unrelated between-subject variance, independence assumed, equal variances assumed). For both phases, the regressors in the design matrix were thus: 0.05€ in 0 days, 0.50€ in 0 days, 1€ in 0 days, 0.05€ in 10 days, 0.50€ in 10 days, 1€ in 10 days, 0.05€ in 100 days, 0.50€ in 100 days, and 1€ in 100 days, followed by one regressor per subject.

One-tailed second-level T-contrasts were then computed. In order to validate our study design, we first aimed to replicate results of previous studies by calculating effects of reward magnitude for both anticipation and win outcomes, comparing the highest with the lowest reward in both phases (1€ > 0.05, contrast: [−1 0 1 −1 0 1 –1 0 1]). Previous studies have shown that higher rewards (compared to lower rewards) elicit higher BOLD-signal in VS during anticipation and higher signal in mPFC (and sometimes VS) during win-outcomes (e.g., Knutson et al., 2001a,b; Hommer et al., 2003), and we thus expected to find the same activations if our design worked as intended. After this validation step, we tested our main hypotheses regarding immediacy and delay of rewards, using the following contrasts: effect of immediacy (0 days > 10 days and 100 days) [2 2 2 −1 −1 −1 −1 −1 −1]; effect of a short vs. a long delay (10 days > 100 days) [0 0 0 1 1 1 −1 −1 −1]; interaction of immediacy with magnitude (stronger effect for 1€ > 0.05€ for 0 days compared to 10 and 100 days): [−2 0 2 1 0 −1 1 0 −1]. Even though we were mainly interested in the outcome phase, for completeness we also calculated these contrasts for the anticipation phase.

We focused our analyses on *a priori* defined ROIs using three bilateral masks: (i) VS; (ii) mPFC; and (iii) amygdala (Table 1; Supplementary Figure S1). Our ROIs served to spatially constrain our analyses, and for the purpose of family-wise error (FWE)-correction for the respective ROI volumes. The voxel-wise significance level was set to *p* < 0.017, FWE-corrected for the ROI volumes (corresponding to a Bonferroni-corrected 0.05, because small-volume FWE correction was applied to each of the three ROIs separately).

### Brain-Behavior Correlations

We then tested for a potential relationship between personality traits (Impulsivity, Anxiety-Depression) and BOLD responses to immediate vs. delayed rewards in the outcome phase. For this purpose, we extracted mean beta values from the regions that showed effects of immediacy at a corrected significance level (either a main effect of immediacy, or an interaction of immediacy × magnitude). To this end, we determined the group peak voxel of the second level contrasts of immediacy or immediacy × magnitude and created a spherical (*r* = 5 mm) ROI centered around this group peak coordinate. Mean beta values were extracted from all voxels located in this sphere for all participants using the *MarsBaR* ROI analysis toolbox.^2^ We then computed the difference between the mean beta over all magnitudes for immediate rewards (0 days) minus the mean beta over all magnitudes for delayed rewards (10 days and 100 days) within these spheres as a neural marker of delay discounting. Next, correlations were computed between this neural measure of immediacy effects and the two factors Anxiety-Depression and Impulsivity (two-tailed testing). Pearson’s correlations were used whenever data were normally distributed, and Spearman’s correlations were used when data did not meet normal distribution.

## Results

### Factor Analysis of the Questionnaire Data

Means and standard deviations of all questionnaires are shown in Table 2. In all participants, scores of the BDI and STAI-T were below clinical cut-off. Our factor analysis identified two factors in our questionnaire data that explained 70.33% of the total variance (Table 3). The first factor, which was termed *Anxiety-Depression* was characterized by positive contributions of NEO—Neuroticism, TCI-Harm Avoidance, STAI-T and BDI and by a negative contribution of NEO—Extraversion. The second factor, henceforth referred to as *Impulsivity*, constituted of high scores in TCI—Novelty Seeking and BIS-11 total and by low scores in NEO—Conscientiousness. To verify the reliability of the thus obtained constructs, we performed the factor analysis in an independent cohort of 125 participants who had completed a largely comparable set of questionnaires. We essentially replicated the results of our factor analysis in that cohort (see Supplementary Table S1).

**Table 2:** **Questionnaire data**.

Questionnaire	Minimum	Maximum	Mean	*SD*
STAI-T	24	60	38.74	9.64
BDI	0	16	4.23	3.82
NEO—Neuroticism	2	37	18.11	7.73
NEO—Extraversion	14	45	31.48	7.35
NEO—Conscientiousness	12	48	30.89	8.23
TCI—Novelty seeking	1	36	22.02	7.45
TCI—Harm avoidance	0	31	11.42	6.91
BIS-11 (Total)	44	95	66.81	9.76

*Descriptive statistics for all questionnaires used in the factor analysis, only for participants who had no missing values and who were therefore included in the factor analysis and in all analyses that involved factor scores (n = 62)*.

**Table 3:** **Pattern matrix from the factor analysis**.

	Component	
	1	2
Questionnaire	Anxiety-Depression	Impulsivity
NEO—Neuroticism	**0.84**	0.16
TCI—Harm Avoidance	**0.84**	−0.24
STAI—T	**0.82**	0.22
NEO—Extraversion	**−0.73**	0.42
BDI	**0.70**	0.23
TCI—Novelty Seeking	−0.33	**0.86**
BIS-11—Total score	0.19	**0.81**
NEO—Conscientiousness	−0.39	**−0.67**

*Factor loadings higher than 0.60 are marked in bold*.

### RTs During Task and their Relation to Anxiety-Depression and Impulsivity

**Figure 2:**
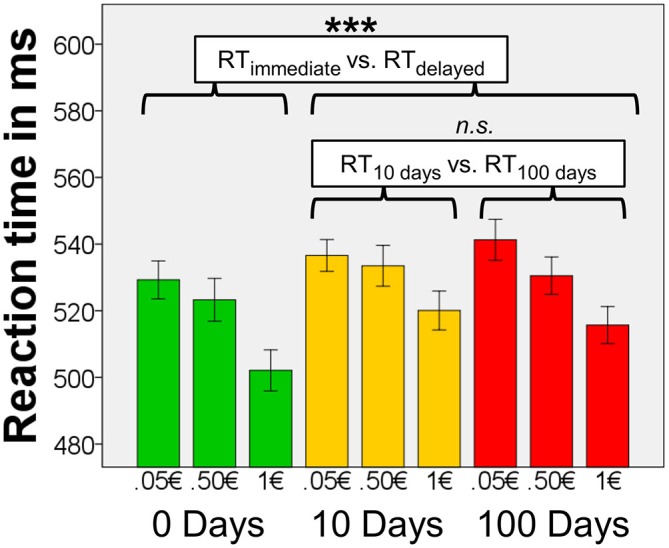
**Reaction times (RTs).** Means of the median RTs per condition (*n* = 72). There were significant main effects of magnitude and delay, but there was no interaction effect of magnitude × delay. Results of the *post hoc* tests concerning effects of delay are shown in the figure. Error bars denote 95% confidence intervals (C.I.) of the mean, adjusted for within-subject designs (Loftus and Masson, 1994). ***p < 0.001.

Median accuracy for all participants on the task was 99.6% (range 92% – 100%). In the analysis of RTs to the target stimuli, we found a main effect of delay (*F*_(1.95, 138.28)_ = 12.97, *p* < 0.001), and magnitude, (*F*_(1.95, 138.51)_ = 46.81, *p* < 0.001), but no interaction of delay × magnitude, (*F*_(3.61, 256.43)_ = 1.12, *p* = 0.35; Figure 2). When including the factor scores Anxiety-Depression and Impulsivity as covariates, there were no main effects of these factors and no interactions between either these factors and delay or magnitude (all *F* < 1.10, all *p* > 0.30), suggesting that neither Anxiety-Depression nor Impulsivity affected RTs. Covarying for smoking status did not change the overall pattern of results.

To follow up the main ANOVA, we carried out four *post hoc* tests to determine the nature of the significant main effects of delay and magnitude (in *n* = 72). We applied a Bonferroni-corrected significance threshold (*α* = 0.05/4 = 0.0125). When testing for the main effect of delay, we found that the mean of the median RTs for immediate rewards were lower than RTs for delayed rewards (10 days, or 100 days, averaged; T_71_ = 4.81, *p* < 0.0001). However the mean of the median RTs for 10-days-rewards and 100-days-rewards did not differ (*T*_71_ = −0.36, *p* = 0.719). This indicates that participants responded fast for immediate rewards, and slower for delayed rewards irrespective of the period of delay. Magnitude of the reward, on the other hand, affected RTs in a value-dependent fashion, namely the mean of the median RTs for 0.05€ were longer than those for 0.50€ (*T*_71_ = 2.84, *p* = 0.006), and RTs for 0.50€ were longer than those for 1€ (*T*_71_ = 6.90, *p* < 0.0001).

### fMRI Results

#### Validation of the Study Design: Effects of Magnitude on BOLD-Signal During Anticipation and Win-Outcome

**Figure 3:**
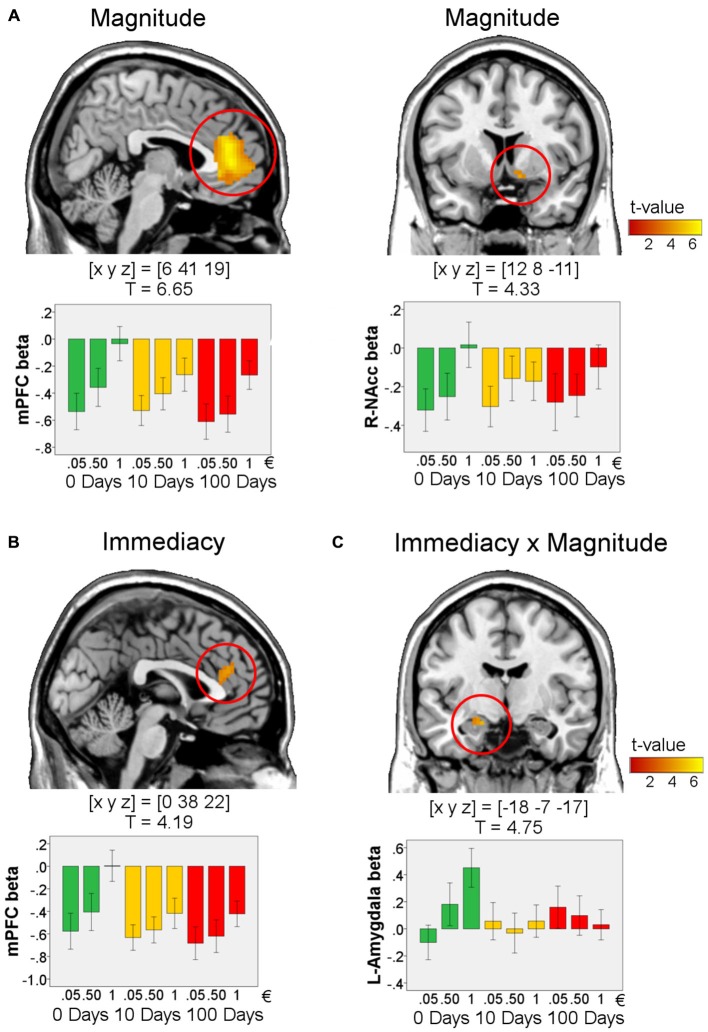
**Effects in the outcome phase for win-trials.** Effects of magnitude (1€ > 0.05€, **(A)**, serves as validation of the study design), immediacy (0 days > 10 and 100 days, **(B)**), and immediacy × magnitude (1€ > 0.05€ for 0 days compared to 10 and 100 days, **(C)**; *n* = 72). For visualization, activations are shown at *p* < 0.001, uncorrected and masked by the respective ROI (medial prefrontal cortex (mPFC), amygdala, or VS). All peaks survive *p* < 0.017, FWE-correction for ROI volumes. T-values and MNI coordinates [x y z] are given for the peaks of the activations. Bar plots visualize the results by showing the mean beta values within a sphere around the peak voxel of each region (radius: 5 mm). Error bars denote 95% C.I. of the mean, adjusted for within-subject designs (Loftus and Masson, 1994).

We first aimed to validate our study design by testing for effects of reward magnitude (1€ > 0.05€) during anticipation and win-outcome. Replicating the results of previous studies, there was an effect of magnitude in the VS [peak in MNI-space [x y z] = [9 8 −2], *T* = 5.38, *p* < 0.0001, FWE-corrected for ROI volume] during anticipation. Further in line with previous studies, during the outcome phase we found effects of reward magnitude in the mPFC [peak in MNI-space [x y z] = [6 41 19], *T* = 6.65, *p* < 0.00001, FWE-corrected for ROI volume] and in the right VS/NAcc [peak in MNI-space [x y z] = [12 8 −11], *T* = 4.33, *p* = 0.002, FWE-corrected for the ROI volume; Figure 3A. These results indicate that our design worked as intended.

#### Effects of Immediacy on BOLD-Signal During the Anticipation Phase

Although the main goal of our study was to assess the neural correlates of delay discounting in the outcome phase, we also analyzed the anticipatory phase of the MID task. However, we found no effects of delay (i.e., immediacy, or 10 days vs. 100 days) or of the interaction of immediacy × magnitude that were significant after correction (*p* < 0.05, FWE-corrected for the ROI volumes). Because there were no robust effects of delay during anticipation, we did not further examine influences of Anxiety-Depression or Impulsivity on brain activation during anticipation.

#### Effects of Immediacy on BOLD-Signal During the Win-Outcome Phase

Immediacy of reward delivery was associated with increased BOLD response within a dorsal portion of the mPFC in response to immediate as compared to delayed rewards (peak in MNI-space [x y z] = [0 38 22], *T* = 4.19, *p* = 0.014, FWE-corrected for ROI volume; Figure 3B). A direct comparison of the BOLD response between delay conditions (10 days vs. 100 days) yielded no reliable differences in the ROIs, even at a very liberal threshold (*p* < 0.01, *k* = 5, uncorrected).

We next tested for interactions of immediacy and reward magnitude. Specifically, we investigated which brain regions showed a more pronounced effect of magnitude (1€ vs. 0.05€) for immediate as compared to delayed rewards. Such a pattern was observed in the left amygdala (Figure 3C; peak in MNI-space [x y z] = [−18 −7 −17], *T* = 4.75, *k* = 10, *p* = 0.00014, FWE-corrected for ROI volume).

#### Correlations of Neural Delay Effects in the Win-Outcome Phase with Impulsivity and Anxiety-Depression

We then tested for a potential relationship between the BOLD response to immediate vs. delayed rewards during the outcome phase and the personality traits of interest (Impulsivity, Anxiety-Depression). To this end, we computed correlations between individual neural immediacy effects (beta_win_immediate_ – beta_win_delayed_) in the mPFC and amygdala (betas averaged in 5 mm spheres around the peak voxel) and the Anxiety-Depression and Impulsivity scores from our factor analysis. Immediacy effects in mPFC were not normally distributed (Kolmogorov-Smirnov *p* = 0.039), while they were normally distributed in the amygdala (Kolmogorov-Smirnov *p* ≥ 0.20). Therefore we used non-parametric Spearman’s correlation for the mPFC analysis and parametric Pearson’s correlation for the amygdala analysis.

**Figure 4:**
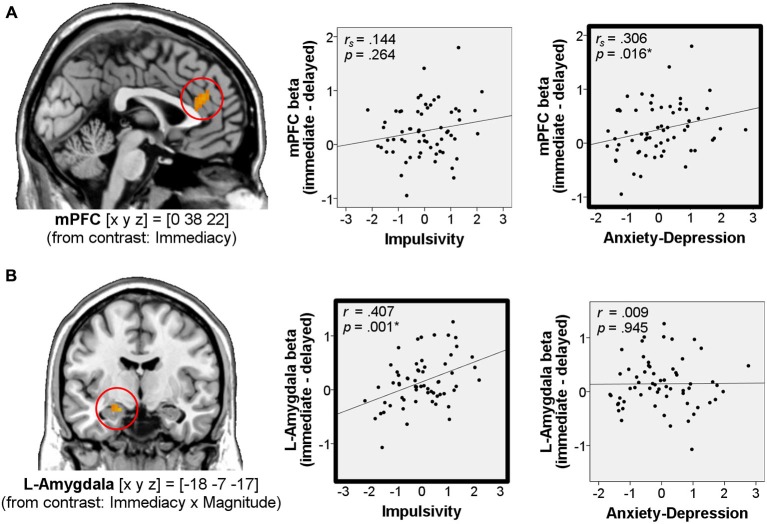
**Correlations of immediacy effects in the win-outcome phase with personality.** Correlations of neural immediacy effects (beta_win_immediate_ – beta_win_delayed_) in the mPFC **(A)** and in the left amygdala **(B)** with the factor scores Impulsivity and Anxiety-Depression (*n* = 62). On the left side of the figure, activations for these regions from the main analysis are shown at *p* < 0.001, uncorrected. Coordinates [x y z] refer to MNI-space. *Significant at *p* < 0.05 (framed in bold).

We found that immediacy effects in the mPFC correlated positively with Anxiety-Depression (*r_s_* = 0.306, *p* = 0.016, two-tailed, *n* = 62, Figure 4A), while immediacy effects in the left amygdala correlated positively with Impulsivity (*r* = 0.407, *p* = 0.001, two-tailed, *n* = 62, Figure 4B).

## Discussion

In the present study, we investigated the neural correlates of delay discounting during reward outcome processing in healthy participants as well as their relationship with individual differences in impulsivity and anxious-depressive traits. We found that a region within the dorsal mPFC exhibited stronger activation for delivery of immediate as opposed for delayed monetary rewards. Moreover, the left amygdala showed an interaction of immediacy and reward magnitude, in that it encoded magnitude only for immediate rewards. Personality traits that had previously been linked to altered delay discounting were found to be associated with the amygdala and mPFC responses in a trait-specific way: While the left amygdala immediacy response was more pronounced in impulsive participants, the immediacy response in the mPFC was more pronounced in participants with anxious-depressive traits.

### Delay Discounting in the Outcome Phase

#### Effects of Immediacy on the mPFC

Previous studies have demonstrated that the mPFC responds to the delivery of predicted rewards during the outcome phase of MID tasks and that it further codes the magnitude of rewards (Knutson et al., 2001b; Hommer et al., 2003; Schott et al., 2007). The present study demonstrates that mPFC activation does not only code the mere magnitude of an outcome, but also its time of delivery: the dorsal mPFC shows a more pronounced response to immediate relative to delayed rewards. Compatibly, Sripada et al. (2011) observed that mPFC activity was higher during decisions that involved one immediate option relative to decisions between two delayed options, although in a somewhat more anterior and ventral region of the mPFC.

The most straightforward explanation of our mPFC finding is that activation in this region reflects the representation or computation of subjective value during the outcome phase. However, as subjective value was not directly measured and as the typical locus of subjective value activations in decision-making paradigms is more ventral (Rangel and Hare, 2010) alternative explanations should be considered. First, our results may relate to the role of the dorsal mPFC in triggering and mobilizing cognitive control (Botvinick et al., 2001, 2004; Ridderinkhof et al., 2004). This process might occur automatically, possibly in order to counteract the tempting nature of immediate rewards (see Fitzsimons and Bargh, 2004). Second, more pronounced mPFC responses to immediate rewards might also reflect increased self-relevance of immediate rewards, since mPFC has been related to self-referential processing (e.g., Gusnard et al., 2001) and also, more specifically, to confirmatory self-referential responses (Sajonz et al., 2010). Third, activation of the mPFC subregion might simply signal immediacy itself (see Sripada et al., 2011).

#### Effects of Immediacy on the Amygdala

An interaction of immediacy and magnitude was observed in the left amygdala, where magnitude was only encoded for immediate rewards. In addition to its well-known role in emotion processing, the amygdala may also, more generally, respond to stimulus salience or relevance (Liberzon et al., 2003; Ewbank et al., 2009; Mahler and Berridge, 2009; Schardt et al., 2010). In line with the notion of the amygdala as a relevance or salience detector, irrespective of valence, individual variability of dopaminergic signaling has been linked to amygdala responses during both reward processing (Schott et al., 2008) and aversive emotional stimulation (Kienast et al., 2008). With respect to our present findings, this could be interpreted as indicating that the amygdala codes an integrated salience or relevance signal that encompasses both magnitude and immediacy of an obtained reward. However, further studies employing both appetitive and aversive reinforcement will be required to systematically address this question (e.g., see Camara et al., 2009).

#### All-or-None Effects of Delay

Neither the mPFC nor the VS or amygdala differentiated between the two delay conditions (10 days vs. 100 days). This was mirrored by the behavioral results of the classification task, in which participants exhibited no significant reaction time differences between rewards delivered in 10 days vs. 100 days, while responses to immediate rewards were significantly faster than those to delayed rewards. This is compatible with the previous observation that the mPFC and the striatum primarily activate during choices involving immediate rewards, showing no further differentiation between rewards delivered after 2 weeks or 1 month (McClure et al., 2004). Our results expand the observation by McClure and colleagues, showing that such a pattern also applies to the valuation phase in isolation. While we cannot rule out that increasing the difference between delays even further (e.g., 5 days vs. 1 year) might ultimately lead to measurable neural differences between the delays (Kable and Glimcher, 2010), our results support the idea that immediacy *per se* is an important factor contributing to the salience of stimuli (see McClure et al., 2004, 2007). Alternatively, or additionally, because participants did not engage in decision-making, they might have—implicitly or explicitly—ignored the difference between the 10-day and 100-day delays.

#### Consideration on the Outcome Phase for Delayed Rewards

Finally, one might note that the outcome phase in this fMRI paradigm did not involve the receipt of an actual reward in the very same moment. It is, of course, logically impossible to receive a reward that will be delivered delayed in time (but see Prévost et al., 2010, for paradigms that involve actually waiting for the delivery of delayed rewards during neuroimaging; Jimura et al., 2013). Rather, our paradigm involved the promise to receive the amount immediately after scanning or following a certain delay after scanning, thus comparing very short to moderate and long delays.

### No Delay Discounting in the Anticipation Phase

In contrast to a previous study that investigated the neural correlates of delay discounting with an MID task (Luo et al., 2009), we found no effects of delay during the anticipation of rewards. Most notably, we could not replicate the immediacy effect in the mPFC during reward anticipation, but only in the outcome phase. One reason for this might be the complexity of our paradigm that included nine different types of reward (three reward magnitudes times three delays), as compared to the (simpler) two-by-two design employed by Luo and colleagues. Thus, in our paradigm, participants might have experienced some difficulty integrating the complex symbolic anticipatory cues in order to form a mental representation of both the magnitude and the delay of the anticipated reward. Instead, they might only have formed a clear representation of the presumably most salient feature, namely the magnitude of the reward during the anticipation, which had strong effects in the VS also in the current study.

One result of our study that speaks for such an incomplete representation of the expected rewards in the anticipation phase is the fact that the VS showed a magnitude effect also during the outcome phase. Previous studies have demonstrated that, in young healthy participants, the VS is activated primarily during the anticipation phase of MID tasks, but not during the outcome phase (Knutson et al., 2001b). This pattern is, however, widely believed to depend on the predictability of rewards (Schultz et al., 2000; Berns et al., 2001; Spicer et al., 2007). In our paradigm, rewards were delivered to correct responses in only 60% of trials. Therefore, the EV of the rewards was reduced during the anticipation phase, while reward delivery during the outcome phase was likely to elicit a positive prediction error (e.g., Pagnoni et al., 2002). With respect to the mPFC, the uncertainty during reward anticipation might have led to the delayed valuation of the rewards to the outcome phase.

It must be noted, though, that during the classification task, participants exhibited shorter RTs to immediate vs. delayed rewards. It can thus not be excluded that neural activation differences during the anticipation phase might not have been picked up due to lack of statistical power, particularly when the variance explanation by the delay effects was small compared to that of the magnitude effects.

### Individual Differences in the Neural Correlates of Delay Discounting in the Outcome Phase

In addition to investigating the neural correlates of the valuation component of delay discounting during the outcome phase, a second aim of our study was to investigate how delay discounting relates to interindividual variability of personality traits that are implicated in neuropsychiatric disorders. To this end, we performed a factor analysis on several well-established questionnaires, namely the NEO-FFI, the TCI, the BIS-11, the STAI-T, and the BDI. Two factors were reliably identified, and considering the contributing variables, they were labeled Anxiety-Depression and Impulsivity (Table 3). With previous studies demonstrating that impulsivity is associated with increased delay discounting (e.g., de Wit et al., 2007; Mobini et al., 2007), and some evidence for a correlation of delay discounting with anxiety-related traits (Rounds et al., 2007), we aimed to correlate the brain activation patterns observed as a function of immediacy with these two traits.

#### Correlation of Impulsivity with Immediacy Effects in the Amygdala

Previous studies investigating the relationship between impulsivity and the neural correlates of reward processing have mostly focused on the response of the striatum. Those studies have yielded partly conflicting results, as both positive (e.g., Forbes et al., 2007) and negative (Beck et al., 2009) correlations between ventral striatal reward responses and impulsivity have been reported. A recent meta-analysis suggests that the relationship between impulsivity-related personality traits and the striatal reward response might depend on the population investigated, with healthy participants showing positive correlations of impulsivity and striatal reward responses, while patient populations with clinically relevant levels of impulsivity may show the opposite pattern (Plichta and Scheres, 2014).

With the focus of the present study being the investigation of delay effects, we did not compute correlations of individual differences with the striatal reward response, as striatal activity was not modulated by delay. Rather we tested for correlations in the amygdala and the mPFC, which both showed effects of immediacy in the outcome phase. We found that the factor *Impulsivity* correlated positively with the response of the left amygdala to immediate vs. delayed rewards in the outcome phase. As discussed above, the amygdala, apart from its well-characterized function in emotion processing, is also believed to convey the signaling of stimulus salience or relevance (Liberzon et al., 2003; Ewbank et al., 2009; Mahler and Berridge, 2009; Schardt et al., 2010). Studies of the neural mechanisms of impulsivity have previously identified the amygdala as an important anatomical structure mediating impulsivity. Volumetric investigations point to a role of the amygdala in motor impulsivity (Gopal et al., 2013), and impulsivity correlates positively with the amygdala response to winning monetary rewards in a slot-machine game (Shao et al., 2013). Our results are in line with the latter finding and extend it by showing that high impulsivity is not only associated with a more pronounced amygdala response to winning rewards in general, but rather, that impulsive individuals are also specifically more responsive to winning* immediate* as compared to delayed rewards. This is in line with most definitions of impulsivity that center around the idea that impulsive individuals are focused on the present and on short-term gratification (e.g., see Evenden, 1999). It also complies with a recent connectionist model of intertemporal choice in which impulsivity was modeled as a reduced response threshold, leading to faster choices and an attenuated influence of the value of delayed rewards (Scherbaum et al., 2012). In summary, our results strengthen the notion that, in addition to the well-known role of the striatum in impulsivity, the preference for immediate rewards in impulsive individuals may at least in part be mediated by the amygdala.

From a clinical point of view, the positive direction of the correlation between immediacy-related amygdala responses and impulsivity is noteworthy, as amygdala hyperactivity has been repeatedly observed in Borderline personality disorder (BPD; Krause-Utz et al., 2014), a psychiatric condition characterized by dysfunctional levels of impulsivity that often lead to self-harming behaviors in the affected patients. Delay discounting is very pronounced in BPD patients (Barker et al., 2015), and future studies should investigate a potential role of amygdala hyperactivity in delay discounting in this patient group.

#### Correlation of Anxiety-Depression with Immediacy Effects in the mPFC

While the positive relationship between Impulsivity and immediacy-related amygdala activity was well in line with our hypotheses, the observation that Anxiety-Depression correlated positively with the mPFC immediacy response was not predicted. As the immediacy effect in the mPFC itself has no satisfying single explanation, as discussed above, the interpretation of correlations within the mPFC remains largely speculative. We will constrain our discussion to two possibilities.

The first explanation relates to the possible role of the mPFC in coding subjective value (Hare et al., 2009; Kahnt et al., 2011; Park et al., 2011). The correlation of mPFC activation with Anxiety-Depression would then suggest that (subclinical) anxious-depressive individuals overvalue immediate and/or undervalue delayed rewards. This is compatible with a cardinal feature of clinical depression, the so-called Beck’s triad (Beck, 1987), which is a pessimistic view of the self, the world and, relevant to this interpretation, the future. It is also in line with findings by Rounds et al. (2007) who reported increased delay discounting in individuals with social anxiety.

A second possibility is that the correlative findings relate to a role of the mPFC in triggering or mobilizing cognitive control in the outcome phase of immediate rewards (Botvinick et al., 2004; Ridderinkhof et al., 2004). In this case the described would mean that anxious-depressive participants trigger or mobilize more cognitive control for immediate rewards, for example because they might ruminate and worry more about receiving—or not receiving—rewards. However, these interpretations are highly tentative at this point and will have to be tested in future, specifically designed experiments.

Future research should expand this approach to populations with clinically relevant levels of anxious-depressive traits, like patients with affective disorders, anxiety or personality disorders like Borderline or avoidant personality disorder. One previous study has suggested reduced delay discounting in patients with social anxiety disorder (Rounds et al., 2007), and several authors have pointed out the role of medial prefrontal and rostral anterior cingulate dysfunction—and possibly hyperfunction—in these disorders (Lemogne et al., 2012; Holtmann et al., 2013; Adhikari, 2014). The observation that anxious-depressive traits correlate positively with the neural immediacy effect in healthy participants points to the possibility that mPFC dysfunction in patients with clinical levels of depression or anxiety might also result in increased delay discounting.

### Subjective Value as an Alternative Explanation

One potential limitation of the present study design is that the effects of immediacy and magnitude might also be, at least in part, attributable to subjective value, as an immediate reward of a certain magnitude has a higher overall subjective value than a delayed reward of the same magnitude. Luo et al. (2009) aimed to circumvent this problem by creating preference-matched stimuli for each participant and could still observe delay discounting effects. In the present study, however, we decided against this approach, because we were interested in individual differences of the valuation process in relation to impulsive and anxious-depressive personality traits. We argued that the correlation of delay discounting effects with decreased overall subjective value is inherent to the phenomenon, since delay discounting is a process of devaluation. Nevertheless, this might constitute a potential limitation of the current study. That is, we can, as of now, not exclude the possibility that, even though we focused on brain structures that responded to immediacy or immediacy-magnitude interactions rather than reward magnitude *per se*, the observed brain-behavior correlations also relate to overall subjective value of the rewards.

## Conclusion

Taken together, our study demonstrates that immediate vs. delayed delivery of rewards engages activity of mPFC and the amygdala during the processing of reward outcome. This pattern of activation is further associated with individual differences in both impulsivity and anxious-depressive traits, with impulsivity correlating positively with immediacy-related amygdala activity, while anxious-depressive traits correlate with immediacy-related mPFC activity.

## Author Contributions

HW and CN designed the study. CN and DW-S carried out the research. VUL and BHS analyzed the data based on and extending earlier analyses by CN, supervised by HW and SE. In collaboration with the other authors, VUL, BHS, and HW interpreted the results of these analyses. VUL, BHS and HW wrote the article, partly based on the doctoral dissertation by CN, and aided by CEW and TG. All authors revised the manuscript critically for important intellectual content.

## Conflict of Interest Statement

The authors declare that the research was conducted in the absence of any commercial or financial relationships that could be construed as a potential conflict of interest.

## Footnotes

^1^www.fil.ion.ucl.ac.uk/spm

^2^http://marsbar.sourceforge.net/
