# Supplementary material for: Delay discounting without decision-making: medial prefrontal cortex and amygdala activations reflect immediacy processing and correlate with impulsivity and anxious-depressive traits
Source: Front Behav Neurosci. 2015 Oct 29;9:280. doi: 10.3389/fnbeh.2015.00280 (PMC4624839; doi:10.3389/fnbeh.2015.00280)
Supplement: Supplementary file 1 [file DataSheet_1.pdf]

Figure S1. Regions of interest (ROIs). For details on ROI generation see Table 1 of the main manuscript.

Table S1. Pattern matrix from a factor analysis of the data from an independent sample (n = 125) for the purpose of replicating the factor structure reported in the article.

	Component	
	1	2
Questionnaire	Anxiety-Depression	Impulsivity
NEO - Neuroticism	**.78**	.16
STAI-T	**.86**	.17
NEO - Extraversion	**-.88**	.22
BDI	**.82**	.08
BIS-11 – Total score	-.09	**.97**
NEO - Conscientiousness	-.24	**-.76**

Factor loadings higher than .60 are marked in bold.

Note. The factor analysis was carried out in the same way as the one described in the main paper, using SPSS 20.0. We included data from the same questionnaires except that no TCI questionnaire data were available for this sample. The factor analysis identified two factors that explained 77.70% of the total variance. The results are very similar to the ones found in the current sample (compare with Table 3 in the main article). Sample used for this replication: N = 125 healthy participants, 63 female, mean age = 25.96, SD = 3.773, range: 20 -35.
