# Supplementary figures and images for: Delay discounting without decision-making: medial prefrontal cortex and amygdala activations reflect immediacy processing and correlate with impulsivity and anxious-depressive traits
Source: Front Behav Neurosci. 2015 Oct 29;9:280. doi: 10.3389/fnbeh.2015.00280 (PMC4624839; doi:10.3389/fnbeh.2015.00280)

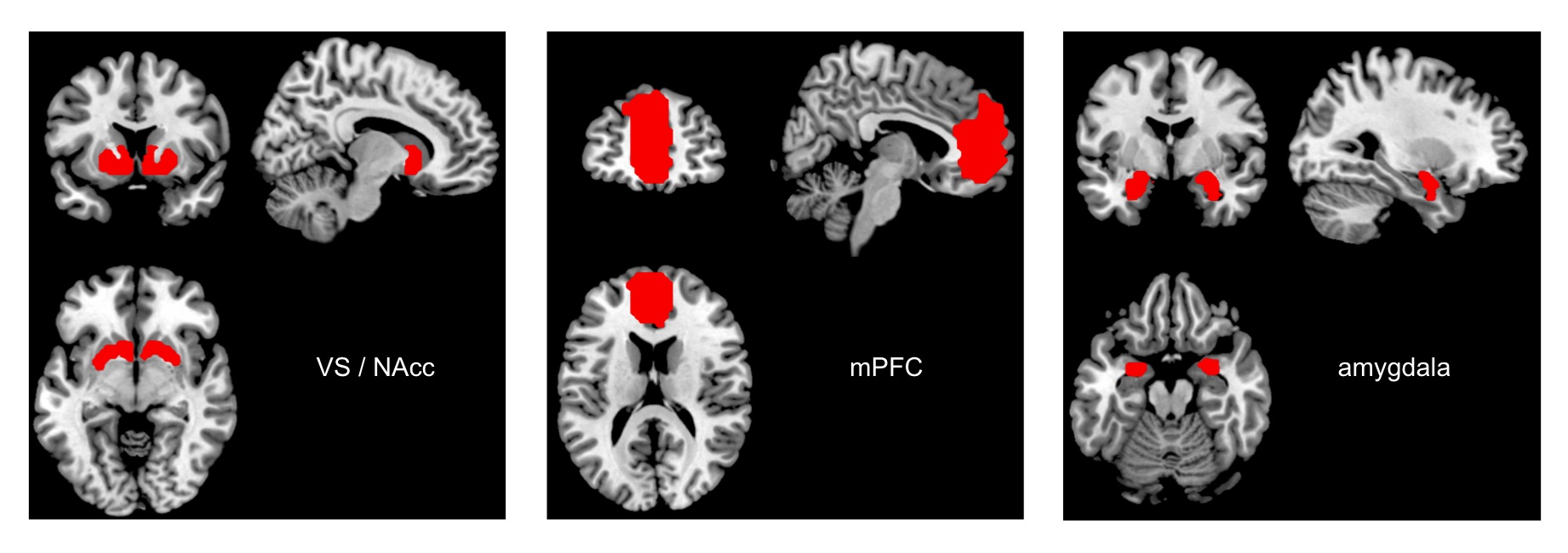

Supplement: Supplementary file 2 [file Image_1.jpeg]
